# The Applied Anatomy of the Nervous System

**Published:** 1888-12

**Authors:** 


THE APPLIED ANATOMY OF THE NERVOUS SYSTEM. * * * By Ambrose L. Ranney, A. M., M. D., Professor of the Anatomy and Physiology of the Nervous System in the N. Y. Post-Graduate Medical School and Hospital. * * * Second Edition re-written, enlarged and profusely illustrated. New York: D. Appleton & Co. 1888.

There has been awakened in the last number of years such a deep interest in the study of the diseases of the nervous system, that a publication giving the results of the most recent investigations in the anatomy and physiology of this system must meet with a favorable recognition at the hands of the medical reading public. A thorough knowledge of this most important subject underlies the successful practice of both surgery and medicine. A reliable guide, therefore, which has for its purpose the accomplishment of this object, cannot but be duly appreciated. From a careful reading of this volume we are able to say that in it we have such a work. While dealing with a subject which has always been regarded as one of the most difficult to elucidate, the writer has presented it in such an interesting shape as to constantly engage the attention of his readers. He is careful on all doubtful points to give the opinions of those who have made this branch of medicine their especial study. The book is replete necessarily with the deductions drawn from the latest researches of the most eminent neurologists and physiologists. Particular attention is bestowed upon the conditions called hemianopsia and aphasia, which play such an important *rôle* in the location of the various lesions and tumors of the cerebrum, and the careful study of which will add much to the making of brain-surgery, which is now in its infancy, a more exact science. The plates are carefully selected, and the diagramatic illustrations well conceived; both of which add much to the comprehension of the subject-matter. We feel well satisfied that the careful study of this volume will repay with profit and pleasure both student and practitioner. W. H. W.

